# Supplementary material for: Piloting competency assessments for an evidence-based brief psychological intervention with Arabic-speaking non-specialists in Switzerland
Source: Glob Ment Health (Camb). 2025 Jun 11;12:e72. doi: 10.1017/gmh.2025.10023 (PMC12277206; doi:10.1017/gmh.2025.10023)
Supplement: Hemmo et al. supplementary material [file S205442512510023Xsup001.docx]

# Supplementary material

# Supplementary S1: PM+ competency assessment items published on the EQUIP platform for flexible use (www.equipcompetency.org)

**Problem management**

1. Recognizing solvable and unsolvable items

2. Selecting the problem

3. Defining the problem and goal

4. Brainstorming solutions

5. Choosing a solution

6. Developing an action plan for the solution

7. Reviewing managing problems

**Stress management & relaxation**

8. Psychoeducation

9. Introduce new technique (then practice & repeat)

10. Check in and encourage continued practice (assign homework)

**Behavioral activation**

11. Scheduling activities and tasks

**Interpersonal**

12. Strengthening social support

# Supplementary S2: WHO PM+ Training Manual Competencies for PM+ competency assessment (in planning)

**Stress management & relaxation**

1. Introducing and explaining slow breathing

**Problem management**

2. Listing and distinguishing solvable and unsolvable problems

3. Choose a problem

4. Define the problem

5. Brainstorming solutions

6. Decide and choose helpful strategies

7. Developing an action plan for the solution

**Behavioral activation**

8. Get going, keep doing

**Interpersonal**

9. Strengthening social support

# Supplementary S3: Actor-Case-Vignette including structured variations

**Role-play ‘ENACT’**

Samer is a 28-year-old man from Syria

- married
- 2 (vs. 3) children (with varying genders throughout the role-plays)
- has been living in a refugee center in (vs. near) Zurich for 5 years
- studied medicine (vs. laborer) before migrating to Switzerland, having a stable life in Syria before the war
- Problems:
  - Stress, anxiety, depression, recurring headache (vs. backache vs. stomachache) when thinking too much about his problems.
  - Financial difficulties
  - Wanting to find a housing outside the refugee camp
  - Feeling ashamed towards family for not having a job matching his diploma
  - Feeling lonely and socially isolated

Samer often feels hopeless and sometimes thinks about suicide, triggered by his constant comparison of his life in Switzerland to his past in Syria, when life was generally better. Due to his contact with a social worker in the refugee camp, he was advised to visit enroll in the PM+ program.

Active prompting during role-play:

- Goal setting (“can you solve my problems?”)
- Future hope (“everything will be solved, and we will live just like in Syria?”)
- Suicidal thoughts (random hopes that all struggles will suddenly stop vs. wish to suicide if things don’t change vs. “do you think, taking a lot of pills would be a good way to suicide?”; no vs. one suicidal attempt in the past; good reasons to live: suicidal thoughts are not serious vs. as a Muslim believer it is forbidden to suicide vs. would not dare to try (again) because of the children)
- Confidentiality (“will you tell someone vs. the camp authorities vs. my wife vs. Caritas about what we discuss in our sessions?”)
- Involving family members in sessions (“could my wife join the PM+ sessions, as she could also benefit?”)

**Role-play ‘Managing Problems’**

Problem 1

Vague problem: Loneliness because of language barrier; Client tried a language course but could not keep on visiting it, because group size was big and the loud noises triggered him, he was not able to concentrate and got irritable. So, he got frustrated and stopped attending.

Solutions:

Silly solution: Shout the group quiet

Unhelpful solutions: Drink alcohol to relax before the course vs. giving up learning German vs. avoiding social interactions where German language competence is needed

Unrealistic solution: Learn through books (vs. hire a private teacher) although he was not able to study on his own before (vs. is lacking money to hire someone)

Realistic solution: Search for a small, free language course.

Action plan: Ask about free, small courses and then find out when, where, and how to join (e.g. via Caritas, friends, social service, social media).

Problem 2

Vague problem: Housing; He is struggling to find a home outside the refugee camp while fearing that current conflicts in the camp might escalate. He feels that causing issues in the camp might bring him attention and increase his chances of being relocated.

Solutions:

Silly solution: Complain loudly to the refugee camp management

Unhelpful solutions: Do nothing, remain in the refugee camp, and assume that no solutions are possible vs. pray and wait, while hoping for change

Unrealistic solution: Apply for private housing despite lacking financial stability or legal eligibility

Realistic solution: Seek help from someone with connections to Caritas, who can

provide housing assistance. If language is a barrier, involve someone to facilitate communication (a trusted friend vs. a social worker from Caritas)

Action plan: Exchange with friends in order to find out who (which friend vs. organization) could effectively assist in this matter. Then, contact this person (vs. this organization) to arrange a personal meeting to discuss this matter.

Problem 3

Note: This problem is only to be listed, but not to be managed in the role-play as the other problems listed are more important to the client right now.

Vague problem: Financial problems; wants to earn more money as fast as possible to support family that stayed in their home country.

**Role-play ‘Managing Stress’**

Obstacles: When the helper asks you about your homework practice, answer that you are feeling unsure about the exercise. You can comment that you have not practiced much on your own at home since the last session. Potential reasons (mention max. two per role-play):

- breathing speeding up when trying
- hard to start it when being already anxious (vs. stressed), then being not effective
- not sure if you’re doing it right
- being too focused on counting the seconds and becoming frustrated when the exercise doesn’t immediately work

**Role-play ‘Review of Managing Problems’**

Obstacles: When the helper asks about implementation of the solution, tell them that you were unable to implement the solution, although you gathered information about free courses (vs. housing options). But you didn't try to call (vs. write), as you were afraid, they wouldn't understand you.

New Action Plan: Ask a friend who speaks German to support you enrolling in the course (vs. calling the social worker for further steps regarding relocation).

**Role-play ‘Review of Get Going, Keep Doing’**

Obstacles to Action Plan 1: When working on scheduling activities, say that you were only able to complete the planned activity (going out to nature) once instead of the three times. Potential reasons (mention max. two per role-play):

- felt too tired
- were worried you might fail in completing the activity in full another time.
- being lazy
- waiting for a friend to accompany
- forgetfulness

New Action Plan: Use of reminders. Plan walking rounds for the start that are short (vs. Plan to agree with a friend on a specific time for a walk together).

Obstacles to Action Plan 2: The client had planned to participate once in a group picnic, organized weekly by Caritas. However, he did not follow through. Potential reasons (mention max. two per role-play):

- a general lack of motivation, “didn’t feel like it”
- forgetfulness
- fear of failing the task
- low energy, being too tired

New Action Plan: Use of reminders. Plan smaller social outings and take gradual steps toward increasing social engagement (vs. ask a friend for company).

**Role-play ‘Review of Strengthening Social Support’**

Obstacles: When the helper asks about Strengthening Social Support, you struggle a bit with who you might depend on. You had intended to reach out to a friend or son for translation to help in contacting organizations for a smaller free German course (vs. the housing topic), but when you did, you felt worried and guilty that you might be bothering them, even though you don’t have any evidence that they did not have time or did not wish to help you.

New Action Plan:

- Ask them directly whether they truly have no problem in helping out
- Explore alternative ways of seeking help, such as involving his wife or another trusted person in the process

**Role-play ‘Staying Well, Looking to the Future’**

When the helper begins to explain the task about Looking to the Future, genuinely indicate whether you understand the activity as just described. If you did not, as their explanations were not sufficient, ask questions to clarify the activity. However, your behavior may appear nervous and worried. If you are asked, you can indicate that you feel a little bit worried, because it indicates the end of the program, and you feel anxious about:

- Needing to implement these strategies in future without the helper’s assistance.
- Fear about potential relapse

Note: This role play will not require you to elaborate on the 'How to Help Others' case example. As soon as the helper introduces this part, the rater will urge the helper to skip it and move on with the session.

# Supplementary S4: Assessment schedule

| **Component** | **Duration in minutes** |
| --- | --- |
| Settle + General Instructions to the Helper | 5 |
| Multi-item Role-Play 'ENACT' | 15 |
| Multi-item Role-Play 'Managing Problems' | 10 |
| Single-item Role-Play 'Review of Managing Stress' | 5 |
| Single-item Role-Play 'Review of Managing Problems' | 5 |
| Single-item Role-Play 'Review of Get Going, Keep Doing' | 5 |
| Single-item Role-Play 'Review of Strengthening Social Support' | 5 |
| Single-item Role-Play 'Staying Well, Looking Forward' | 5 |
| Exchange between Rater & Actor in absence of Helper | 3 |
| Competency-based Feedback from Rater to Helper | 7 |
